# Enriching Mental Health Mobile Assessment and Intervention with Situation Awareness †

**DOI:** 10.3390/s17010127

**Published:** 2017-01-10

**Authors:** Ariel Soares Teles, Artur Rocha, Francisco José da Silva e Silva, João Correia Lopes, Donal O’Sullivan, Pepijn Van de Ven, Markus Endler

**Affiliations:** 1 Graduate Program in Electrical Engineering, Federal University of Maranhão, 65080-805 São Luís, Brazil; fssilva@lsdi.ufma.br; 2 Centre for Information Systems and Computer Graphics, Institute for Systems Engineering and Computers, Technology and Science, 4200-465 Porto, Portugal; artur.rocha@inesctec.pt (A.R.); jlopes@fe.up.pt (J.C.L.); 3 Department of Informatics Engineering, Faculdade de Engenharia da Universidade do Porto, 4200-465 Porto, Portugal; 4 Department of Electronic and Computer Engineering, University of Limerick, V94 T9PX Limerick, Ireland; donal.osullivan@ul.ie (D.O.); pepijn.vandeven@ul.ie (P.V.d.V.); 5 Department of Informatics, Pontifícia Universidade Católica do Rio de Janeiro, 22453-900 Rio de Janeiro, Brazil; endler@inf.puc-rio.br

**Keywords:** mobile mental health, situation awareness, ecological momentary assessment, mental disorder treatment, fuzzy logic

## Abstract

Current mobile devices allow the execution of sophisticated applications with the capacity for identifying the user situation, which can be helpful in treatments of mental disorders. In this paper, we present *SituMan*, a solution that provides situation awareness to *MoodBuster*, an ecological momentary assessment and intervention mobile application used to request self-assessments from patients in depression treatments. *SituMan* has a fuzzy inference engine to identify patient situations using context data gathered from the sensors embedded in mobile devices. Situations are specified jointly by the patient and mental health professional, and they can represent the patient’s daily routine (e.g., “studying”, “at work”, “working out”). *MoodBuster* requests mental status self-assessments from patients at adequate moments using situation awareness. In addition, *SituMan* saves and displays patient situations in a summary, delivering them for consultation by mental health professionals. A first experimental evaluation was performed to assess the user satisfaction with the approaches to define and identify situations. This experiment showed that *SituMan* was well evaluated in both criteria. A second experiment was performed to assess the accuracy of the fuzzy engine to infer situations. Results from the second experiment showed that the fuzzy inference engine has a good accuracy to identify situations.

## 1. Introduction

Mobile devices such as smartphones and tablets have resulted in an almost continuous connectivity with the world-wide-web and the ability to access, gather, process and share vast quantities of data regardless of time and place. They are equipped with a variety of sensors (e.g., proximity, magnetometer, accelerometer, temperature, humidity) that make them ideal for the gathering of information about user activities and the environment [1]. This, in turn, allows the development of sophisticated applications that correlate multiple pieces of this information to obtain an enhanced understanding of the current user status, context and behaviour. The new and better insights about the behavioural aspects influencing people’s health status thus obtained from mobile devices [2] are relevant to understanding which factors motivate people to change their behaviour to a healthier lifestyle.

The advent of mobile technologies has also caused healthcare to undergo a major transformation. Mobile technologies can be used to monitor personal health information in real time, to access data related to healthcare interventions remotely and to allow access to healthcare resources, such as the establishment of communication channels with physicians and other health professionals. Access to health services can be limited by factors, such as low income, the availability of insurance, time or because of social factors, such as the stigma associated with mental health conditions. The sense of privacy associated with mobile-based services makes them especially appealing to deal with this stigma. Mobile technologies not only allow continuous monitoring of an individual’s physiological and psychological state, but also contribute to build up a lifelong record of physical, mental and social health. Hence, the use of information and communication technologies in healthcare interventions, also called e-health, may be able to mitigate the trend of ever-growing healthcare costs whilst improving access to health services.

In recent years, mobile devices have been applied to the area of mental health as part of medical, psychological and general health services in order to aid in the treatment of mental disorders [3]. Mental disorders include, for example, alcohol and drug use disorders, mood disorders (e.g., depression) and delusional disorders. The use of mobile devices can be effective in the treatment of mental disorders by: (1) allowing ecologically-valid information from users through momentary assessments; (2) providing patients with real-time feedback about behavioural patterns and tips on how to change them; and (3) providing remote support [3]. The long-term focus of the work presented in this paper is to enable the provision of the aforementioned three points in a more meaningful way by carefully assessing the user’s current context and scheduling aspects of the treatment accordingly. Two major issues in this regard are:

(1) How does one accurately assess the patient’s mental status by minimizing the recall bias and, at the same time, not being excessively intrusive? Recall bias is a systematic error due to differences in the accuracy or completeness of the recall of past events or experiences. The memory bias alters recalled memories of past events, introducing significant differences to what actually happened to the patient. This poses problems in a psychological treatment where questions are posed by the mental health professional (e.g., psychologist or psychiatrist) to the patient several days later.
(2) How does one accurately identify situations experienced by the patient in a time period? Having a better insight into the patient’s daily routine, a mental health professional may be able to discuss the causes and effects of depressive symptoms (e.g., “if they are spending too long at work”; “if they are sleeping for most part of the day”), thus improving the effect of therapy. Moreover, the professional can correlate situations experienced by patients with the record of their assessments, thus trying to infer which situations have a positive or negative impact on the patient’s state of mind.

In this work, we present Situation Manager (*SituMan*), which aims to resolve the aforementioned two issues. *SituMan*, parts of which were previously reported in [4], consists of a mobile system that provides situation awareness [5,6,7] to mobile applications, such as the ones in the scope of mental disorder treatments. In *SituMan*, a situation expresses and represents daily routine states named by the user, such as “working”, “studying”, “at mother’s house”, “socializing”, etc. *SituMan* uses a fuzzy rule-based inference engine to infer the user situation from the contextual information obtained from sensors embedded in mobile devices and specified by the user. In this paper, we describe the technical aspects of *SituMan* and its integration with an existing self-assessment application called *MoodBuster* [8], and we investigate the user experience in a pilot feasibility study.

*SituMan* is composed of an Android mobile application and a shared service with a well-defined Application Programming Interface (API) used in the scope of this work to provide situation awareness to *MoodBuster*. The *SituMan* mobile application is used: (1) to express patient situations of interest for the treatment of depression; (2) to identify when the situations happen; (3) to provide the handling of situations available to *MoodBuster*; and (4) to save all changes of the situation in a summary. With the context and situation information provided through the integration of *SituMan*, we expect that participants will be able to respond to *MoodBuster* questions truly “in the moment”. Therefore, by using situation awareness provided by *SituMan*, *MoodBuster* requests for mental status self-assessments are performed in pre-set situations deemed adequate for the question at hand. In addition, *SituMan* shows patient situations in a summary, delivering them for consultation by mental health professionals.

This paper is organized as follows. Section 2 gives an overview of the area of ecological momentary assessment and intervention, introduces *MoodBuster* in which *SituMan* was integrated and reviews the related work. Section 3 shows the design of the fuzzy inference engine used for identifying daily routine situations in *SituMan*. Section 4 presents the details of *SituMan*’s main implementation issues, highlighting the tools proposed for mental disorder treatments. Section 5 presents two experimental evaluations performed with the proposed solution. Finally, in Section 6, conclusions are drawn.

## 2. Preliminaries and Related Research

### 2.1. The MoodBuster Ecological Momentary Assessment and Intervention

Ecological Momentary Assessment (EMA) is a mechanism used to prompt individuals, at fixed or random times, to respond to questions about what they are doing (or have done) and/or are experiencing (or have experienced), repeatedly, throughout a period of time within their daily routine. According to Shiffman [9], EMA aims “to assess the flow of experience and behaviour over time, capturing life as it is lived, moment to moment, hour to hour, day to day, as a way of faithfully characterizing individuals and of capturing the dynamics of experience and behaviour over time and across settings”. Whereas EMA focusses on obtaining information from the patient as it is experienced by the latter, Ecological Momentary Intervention (EMI) “provides a framework for treatments characterized by the delivery of interventions to people as they go about their daily lives” [10]. Both mechanisms are ecological because they occur in the natural environment and are momentary by requesting self-assessments and providing real-time support in the patient’s everyday life.

EMAs and EMIs (EMA/Is) have been implemented in mobile technologies by using phone calls, text messages and, more recently, mobile applications. Especially the latter are of great interest, as they allow the reporting of experiences and mental status (e.g., mood, motivation, anxiety, sleep quality) in real time, in real-world settings, over time and across contexts/situations. EMI mobile applications have been used to: motivate the patient, encourage engagement in practising or the use of previously-learned skills, aid in the development of new skills, notify or distract individuals when they are at risk of engaging in addictive behaviour and provide individuals with personalized summary data. However, to-date, the main trigger for these EMAs is a pre-determined (or (pseudo-)random) time. Hence, EMA/Is are not typically shown to the user at the most appropriate moment in time as determined by the availability of the user, the need for particular information or support or having experienced an event of interest in the near past.

**Figure 1:**
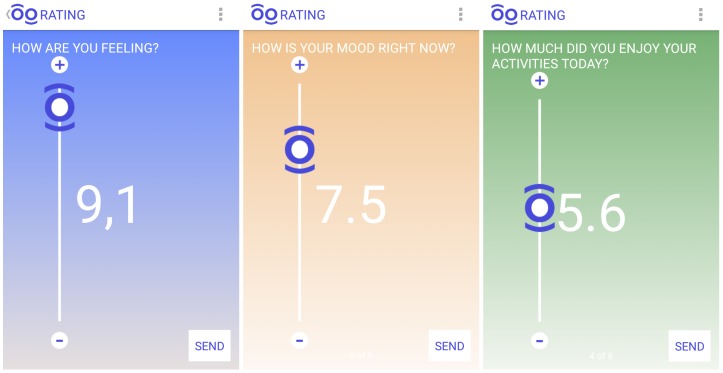
*MoodBuster* mobile GUI for requesting patient self-assessments.

*MoodBuster* is an EMA/I mobile application for use in the treatment of depression. A person can use it to get an accurate view of the way in which his/her mood changes over time. It asks questions about the patient’s state at various moments of the day, i.e., patients are assessed by repeatedly reporting their experiences and mental status (e.g., mood, motivation, anxiety, sleep quality) in real time throughout the day. It also presents accumulated answers in a graph, which can later be consulted by the mental health professional. Such graphs may also give patients a better insight into their own behaviour and symptoms. Figure 1 illustrates the *MoodBuster* interface examples used for requesting self-assessments.

*MoodBuster* was developed in the scope of the ICT4-Depression (http://www.ict4depression.eu/) [11] and E-Compared (http://www.e-compared.eu/) [12] projects. These projects intend to provide mental healthcare stakeholders with evidence-based information and recommendations about the clinical and cost-effectiveness of blended depression treatment. Blended treatment entails a combination of: (1) Internet and mobile based; and (2) face-to-face (traditional) assessments and interventions. The central assumption of the projects is that the two forms of treatment will lead to similar clinical improvements in patients, but that the blended form can be offered at significantly lower costs, by using information and communication technologies.

### 2.2. Related Work

Related work in context-aware mobile computing solutions to support remote monitoring of health in general is vast and varied [13], as is that focused on context information reasoning for recognising human activity/movement [14,15]. Expert systems with context-aware services have also been proposed in the literature to support healthcare, including the adoption of fuzzy logic [16] and the use of sensors for recognizing user situations [17] in order to help the decision making process of health professionals and caregivers.

There is a growing interest in using mobile technologies to unobtrusively monitor depressive patients [18] and to support treatments of mental disorders [3]. For example, *Mobiletype* [19] and *Mobile Therapy* [20] are mobile applications used to monitor and to assess people’s everyday experiences, such as moods, emotions, locations, activities, companionship, stress and alcohol and drugs use. *FOCUS* [21] and *Health Buddy* [22] are more recent mobile systems focused on the treatment of schizophrenia. All four solutions log the time (i.e., the time-stamp) of all user interactions in order to know when they occurred.

*Empath* [23,24] is a remote monitoring system for use in home, which combines context information obtained from sensors embedded in a mobile device, as well as sensors throughout the home and patient self-assessments to detect early signs of mental diseases. Additionally it provides information about the effectiveness of treatments. *Mobilyze!* [25] is a system focused on the treatment of depression, where the mobile application transmits context data to a server to infer relevant information about the patient. The *MONARCA* wearable system [26,27,28] aims to recognize the early warning signs of manic and depressive episodes to predict and prevent their occurrence in a timely manner and to adjust the treatment. This system has a mood forecasting component used for mood prediction.

*StressSense* [29] is a mobile solution concerned with identifying problematic mental health situations, which proposes a methodology for recognizing the occurrence of stress from human voice samples. *MoodRhythm* [30] is another mobile application used to track the patient’s daily routine. It uses context information to estimate the time that the patient spent sleeping and social activity patterns and sends this information to clinicians who provide feedback to patients about their routine to enable them to improve their mood.

*EmotionSense* [31] is a framework proposed for using context awareness in social psychology studies. By processing context information, it has resources for inferring user’s emotions, proximity interactions, conversation patterns and identifying activities. Similarly, *BeWell* [32] is a mobile application developed to promote improved behavioural patterns via user feedback. It also infers important information about the patient, such as sleep duration, physical activities and social interactions. *StudentLife* [33] extends *BeWell* by collecting more types of context data, and it is used to assesses the day-to-day activities of students at a college.

*SituMan* contributions: All presented related work deals with patient mobile remote monitoring using contextual information as an additional resource for improving or reinforcing mental disorder treatments and diagnosis. Our proposal differs by exploring the use of situation awareness as the pass-through mechanism for adaptive self-assessment requests, meaning that the patient may choose to receive (or avoid receiving) specific types of self-assessment prompts according to his or her situation. This strategy is thought to be beneficial for adherence to the EMA requests and for the validity of information provided through these requests. It is worth highlighting that (1) the situation inference process executed by the fuzzy rule-based engine is performed only in the user’s mobile device without requiring any communication with a cloud/server side; this is different to many other mechanisms for situation inference that are based on costly algorithms in terms of computational resources requiring the sensors’ raw data to be exported to a cloud/server infrastructure where the inference process is performed; (2) *SituMan* can provide situation awareness to other local mobile applications, by means of a shared service and a well-defined API, thus making it usable in different application scenarios; and (3) *SituMan* adopts the paradigm of situation awareness, which is relevant in depression treatments by providing helpful information to the mental health professional about the patient’s daily routine.

## 3. The Situation Inference Engine of the SituMan

The inference engine used in *SituMan* was developed based on the following main requirements.

Patients, with help from their mental health professional, must be able to easily express their situations in the mobile application. To this end, the patient should be able to use context information relating to location, time and user activity as determined from the device’s accelerometers to define situations. It is important that these situations are relevant to the mental health professionals in conducting depression treatments, but it is equally important that these situations are defined together with the patient, as the semantic representation of a situation can have different meanings for two different people.

The engine must not predetermine a limited set of situations to be identified and used in treatments. A situation can be considered of interest for the treatment of one patient, while the same situation may not be of interest for another patient or the description of the situation may evolve over time. Hence, the engine must allow the maintenance (adding and removing) of the situations of interest by the user.

The engine (and the mobile application) must work in a “plug and play” form. The engine and the mobile application that uses it must require limited user effort for configuration prior to use.

The engine must identify the patient’s current situation correctly and in real time.

A fuzzy inference engine used to identify the patient’s situations was implemented, along with a service to provide situation awareness to *MoodBuster*. This situation inference engine uses fuzzy logic [34,35] as the basis for identifying situations.

### 3.1. Reasons for Using Fuzzy Logic

Situation identification techniques abstract low level context data into more meaningful high level contexts. Many solutions for understanding context [36] and identifying situations from context data in ubiquitous environments [37] have been developed and proposed in the literature. According to Ye et al. [37], they can be classified into: (1) specification-based techniques, which consist of defining specifications that represent knowledge in logic rules and apply reasoning engines to infer situations from inputs (e.g., logic programming, ontologies and fuzzy logic); and (2) learning-based techniques that use machine learning and data mining to explore association relations between sensor data and situations of interest (e.g., Bayesian networks, decision trees, neural networks and support vector machines). We decided to use a specification-based technique to identify patient daily routine situations because of the following motivations:

(1) Fuzzy logic uses rules that, on a high level, can be defined by non-expert users. In *SituMan*, these rules consist of information on location, time and activities. Hence, by defining a situation or context as a combination of location, time and certain activities, users develop rules for the triggering of EMAs that are transparent and provide end-users with the knowledge when rules will be triggered. This in turn leads to the ability to tailor interventions easily and to a feeling of empowerment in users;
(2) There is no need for a training phase as would be required if any supervised machine learning technique were used, because users define their situations using specifications, such as fuzzy rules (i.e., users perform the role of experts in the system). The use of supervised machine learning for identifying situations would require that each user goes through an individual training phase that could be very time consuming and that could depend on the user providing constant feedback. This individual training phase is required because the context data used to define a given situation experienced by different users are typically different. For example, the situation “working” experienced by Alice is different from that experienced by Bob. In addition, changes in the user’s daily routine would require a new training phase in order to learn the new situations experienced in that new routine.

Of course, a disadvantage of a specification-based situation identification technique is that it will not be possible for users to specify all of the situations that they will confront in their daily lives. However, the *SituMan* objective is to identify only those situations that are relevant to the treatment of mental disorders, i.e., those that the mental health professional is concerned with and the patient agrees to disclose.

There are various specification-based techniques [37]. We have chosen fuzzy logic because of the following reasons [38,39]:

(1) Fuzzy rule-based systems use IF-THEN rules, a deductive form to express inference [39]. This fuzzy inference is the application of decision making structures, called fuzzy rules, using fuzzy logic values (i.e., linguistic variables) and logical connectives. Linguistic variables have values that are not numbers, but words or sentences expressed in a natural or artificial language [40], and these variables can represent imprecise and qualitative human knowledge and are used to label fuzzy sets [38]. Therefore, fuzzy logic provides a notation to represent the inference process using rules that can be easily understood. In other words, fuzzy rules provide a language that allows the user to express contextual information that can represent values as terms instead of using crisp sets, and these terms can be represented in friendly user interfaces.
(2) As described in the literature about the quality of context [41], contextual information obtained from sensors embedded in mobile devices may not be of high quality, such as precision and accuracy. For example, geographic coordinates obtained from a GPS can be imprecise. To cope with this issue, fuzzy logic enables approximate reasoning to conclusions ranging from false to true, i.e., partly true (or partly false).
(3) Situations correspond to a reality that people perceive, live and reason about [42]. This human reasoning is naturally ambiguous, imprecise and qualitative. To cope with these issues, fuzzy logic has been used to computationally model human reasoning [34], such as a real-life daily routine situation. Such endeavours have shown that fuzzy inference systems enable human specialists in a domain to map their experience and their decision making process to computer systems using fuzzy rules.
(4) Fuzzy logic does not require much computing power from the mobile device to perform the inference process, thus avoiding a high demand on battery power or communication with a server/cloud to offload computations. Hence, the identification process of the current user situation does not depend on communication channel conditions or server/cloud availability. This is important because the current user situation is required in real time.

### 3.2. Conceptual Model of the Engine

**Figure 2:**
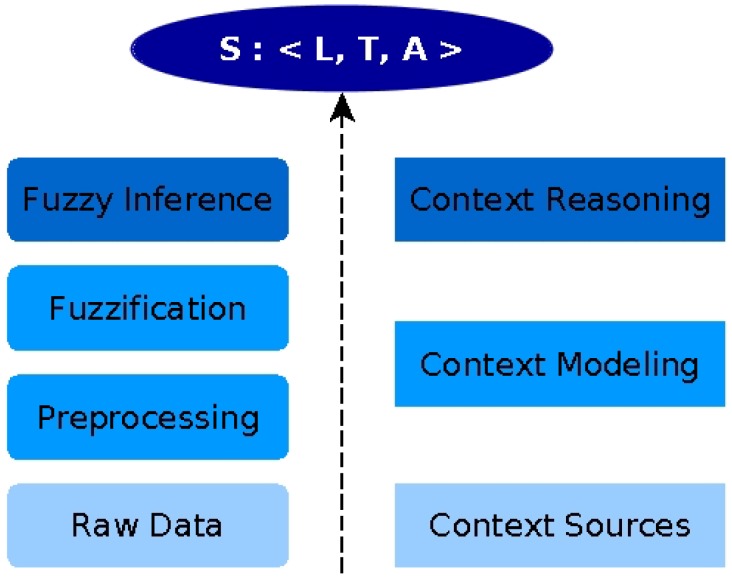
Conceptual model of user situation inference using fuzzy logic.

In *SituMan*, a situation is defined as a tuple *S*: <*L, T, A*>, where *L* represents the current user location (where), *T* is the time represented by “day of the week” and “time of the day” (when) and *A* represents the current user activity (what). From this definition, a situation is identified from the correlation of the context data using a fuzzy inference engine. Figure 2 shows a conceptual model of the *SituMan* situation inference process using fuzzy logic. In this model, context data related to location (*L*), time (*T*) and activity (*A*) are obtained directly from the user mobile device. After collection, the data are preprocessed to adjust their format, e.g., extract redundant or unused information, such as the seconds in time-stamps and the altitude in geographical coordinates.

In the fuzzification phase, context information is represented by linguistic variables. Therefore, data about location, time and activity are represented as fuzzy sets [35]: *L = {same place as a user-chosen location in a map, near that chosen location, different/distant place from the one chosen by the user}*, $T_{time}$ *= {dawn, morning, afternoon, night}*, $T_{week}$ *= {weekday, weekend}* and *A = {during activity}* (i.e., the user performing activities of interest, such as walking, running or even being still). Finally, in the fuzzy inference phase, the different types of context information are then combined, correlated and fused to calculate a user situation.

The conceptual model is flexible, allowing one to add (or remove) contextual information to define and compose a situation. Therefore, it is not restricted to the context data used by the current version of *SituMan*. To use a new type of context data, one needs to model it in a fuzzy set and then generate a new inference engine to be loaded in the application.

### 3.3. Adapted Fuzzy Inference Process

**Figure 3:**
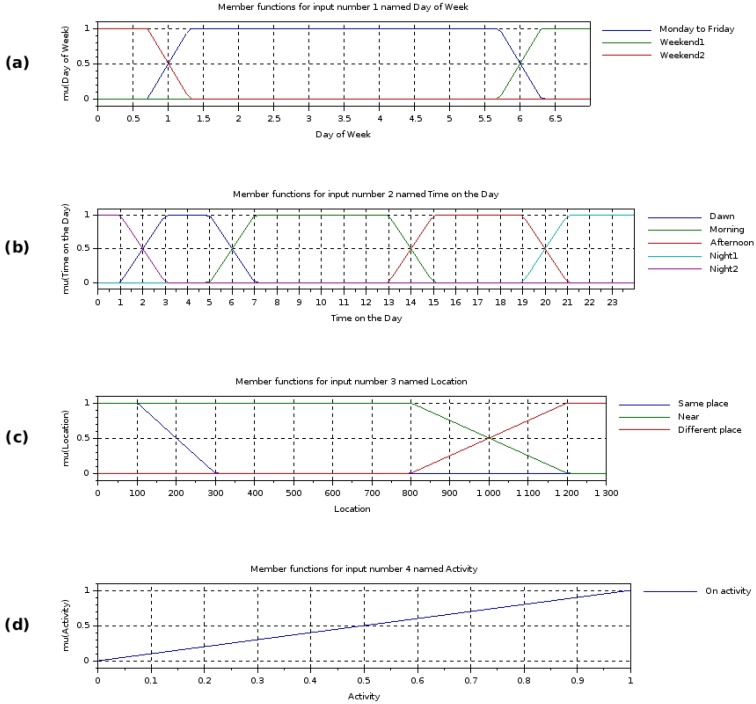
Membership functions of the context information. (**a**) Day of Week; (**b**) Time on the Day; (**c**) Location; (**d**) Activity.

In the conceptual model, a situation is represented by inference rules and fuzzy sets that model context information regarding location, day of week, time of day and activity implemented in the *SituMan*, as illustrated graphically in Figure 3. Fuzzy rules that represent situations are created at run time, i.e., *SituMan* enables the management (addition and removal) of the fuzzy rules (the knowledge base) through the use of the situations’ definition interface. A knowledge base in a fuzzy system is usually static and created by a specialist in the application field, but in *SituMan*, it is maintained by the mental health professional and patient during face-to-face sessions. In this way, they provide the information necessary for building the fuzzy rules that represent the patient daily routine situations.

The ranges of values used in the fuzzy sets of context information have been defined and refined based on the results obtained in practical tests with the application performed by researchers associated with our research labs. In *SituMan*, we decide to use the product method, also called max-product composition [38], to evaluate the situation fuzzy rules. This method is equivalent to the product of the truth values from each condition in the antecedent of a rule. It was chosen because the truth values of all context data in a rule influence the degree of activation of the rule.

Context information regarding time is expressed using fuzzy sets in a cyclic form: from 0 to 6.99 in day of the week (Figure 3a), where each number represents a day of the week, and from 0 to 23.99 in the case of time of day (Figure 3b), where each number represents an hour of the day. As the functions for these sets had to be defined across the limits of a day (from 00:00 to 23:59) and of a week (from Sunday to Saturday), we had to create two sets: “Night1” and “Night2”, as well as “Weekend1” and “Weekend2” , which overlap and which represent each a single linguistic variable, night and weekend, respectively, as shown in Table 1. Therefore, the truth value for weekend is obtained from max(Weekend1,Weekend2) and for night from max(Night1,Night2). For example, if the user creates a situation using “night” to express time of day, the situation inference engine sets the condition of the fuzzy rule to become: **if** time of day is “Night1” **or** “Night2”. Hence, both sets are considered when checking a situation fuzzy rule, which guarantees that the rules are correctly evaluated.

The location information (Figure 3c) is represented as a fuzzy set relative to a point (a geographic coordinate) registered by the user in a map when defining a situation, and the user is considered to be at the same place of the registered point if his/her current Euclidean distance is less than or equal to 100 m from the point. The truth value of this information linearly decreases as the current user position goes from 101 to 300 m from the registered point, where the truth value reaches zero. Furthermore, the user is considered to be near the registered place if he/she is currently located up to 800 m away from the point provided in the situation definition. The truth value of this information decreases from 800 (800 not included) to 1200 m, where it reaches zero. Finally, the user is considered at a different place if the current location is more that 1200 m away from the registered point. The truth value increases from 800 to 1200 m, where it reaches one.

The fuzzy set named “same place” is a subset of the set named “near”. Hence, the first set is used as a delimitation of the second one. That is, a user that is in the same place in relation to a point on the map is also nearby. This was a design decision taken with the goal of modelling the way that people often reason. However, this modelling can cause “cases of doubt” for the engine, in which fuzzy rules can have equal degrees of activation. Consider two situation fuzzy rules defined identically, except for the location information, in which the fist rule is defined with the set “same place”, and the second one uses “nearby”. If the input value for both rules is from 0 to 100 m, then they will have the same degree of activation. In order to deal with this issue, the engine generates a specific exception when the second rule is defined, giving an opportunity for it to be changed. On the other hand, the second rule can be kept, and as in the aforementioned case, situations will be identified simultaneously.

For the case of context information regarding the user’s activity (Figure 3d), only one set named “on activity” is used to represent that the user is performing the following activities specified in the situation configuration: in vehicle, on bicycle, on foot, running, still, tilting and walking. Human activity/movement can be detected using various recognition solutions [14,15]. We have chosen to gather it using the Google Play Services using the activity recognition API (https://developers.google.com/android/reference/com/google/android/gms/location/DetectedActivity), which returns a value from zero to 100 indicating the likelihood that the user is performing an activity (i.e., a probability value). This means that larger values indicate that it is likely that the detected activity is correct. It is important to note that: (1) “on foot” is a generalization of “walking” and “running”; and (2) some activities are not mutually exclusive, e.g., a user can be walking while in a subway. Therefore, the sum of the probability values of all detected activities does not have to be ≤100.

The input value to the activity fuzzy set is calculated from the sum of the normalized probability values of the activities defined by the user in the situation. That is, the input value is obtained from the following function: $\sum_{i=1}^{7}\left(\alpha *\delta \right)$, in which *i* is the activity identifier (there are seven possible activities), *α* is the normalized probability value for an activity that is happening (which is obtained from the activity recognition API), and *δ* is 1 for activities defined by the user in the situation, or 0 for not-defined ones. Therefore, the user can select more than one activity to happen in a given situation. Of course, if more activities are selected in the situation configuration, the chances of the fuzzy rule being activated are higher. For example, when the user configures a situation specifying “walking” and “still” activities to express that both levels of activity are possible in the scope of that situation, the chances of this rule being activated are higher than if the user had only used “walking”. The threshold for the input value used in this fuzzy set is one, even though the sum of the probability values of all detected activities is >100 (i.e., one in a normalized form).

It is important to note that the activities named “still” or “tilting” are also considered types of context information regarding activity, although they do not denote that the user is in motion. Hence, the activity information is not related to context information about the displacement intensity of the user. Instead, it relates to a pattern of movements or the absence thereof. That is, the activity named “on vehicle” is not considered a greater value in the “on activity” fuzzy set than “still”.

The engine can have 24 different rules using these context information fuzzy sets to compose the precedent of rules: three (three different types of locations) * four (four different time periods of a day) * two (weekday and weekend) * one (there is only one fuzzy set of activity) = 24. However, in *SituMan*, the evaluation of fuzzy rules is different to that made by traditional fuzzy systems, because the rules are individually evaluated. That is, the degree of activation is calculated individually for each rule considering the context information specified in the situation defined by the user. This adapted approach to evaluate rules allows input values in the precedent of rules to be different in each individual rule evaluation. More specifically, input values regarding location and activity are determined in each new rule evaluation: (1) the input value for the condition related to location can change very often, because the value obtained from the Euclidean distance between the user and a point on the map registered in a situation can also change (i.e., situations usually are defined for different places); and (2) similarly, the input value for the condition related to activity can change, because a rule can be created with different activities (including more than one activity). In this sense, *SituMan* can use similar rules (with different context information, but using equal fuzzy sets) to refer to different situations. In this way, it allows users to create a large number of situations just requiring the specification of different locations and/or activities. For example, two situations are defined with the same fuzzy sets: “Same place” for Location; “Monday to Friday” for Day of Week; “Morning” and “Afternoon” for Time on the Day; and “On Activity” for Activity. The first situation is named “work”, which is defined using a location (a point on the map) related to a workplace, while the second one is called “leisure” by the user, which is created with another defined place (e.g., the gym where the user trains or the pub where he/she usually goes). Hence, they will even have different input values for location information that has equal fuzzy sets (the “Same place” fuzzy set), because the Euclidean distance between the user and the referred places will be different. Therefore, in the adapted approach to process fuzzy inference, rules using equal fuzzy sets to compose their precedents can have different degrees of activation. Hence, much more than 24 situations can be represented in *SituMan*.

### 3.4. Situation Examples

As mentioned earlier, fuzzy inference systems are based on rules expressed in natural language, and some examples of situation rules that the patient and his/her mental health professional can define are:

(1) **if** the patient is located at home **and** the time of day is in the morning **and** it is a weekend day **and** the patient is still, **then** the situation is *relaxing time*;
(2) **if** the patient is located near his/her workplace **and** the time of day is in the morning **or** afternoon **and** it is a weekday (Monday to Friday) **and** the patient is still **or** walking, **then** the situation is *working*;
(3) **if** the patient is located near the beach **and** the time of day is at night **and** it is a weekday (Monday to Friday) **and** the patient is walking **or** running, **then** the situation is *physical activity*.

Considering the second situation, the correspondent fuzzy rule is evaluated by the the inference engine as follows:

- Location: The user registers a point on a map and determines the linguistic variable “near”. For this example, we will assume a point at his/her workplace. At the time of inference, the engine: (1) checks the current patient location; (2) checks the coordinates recorded in the situation (i.e., “user’s workplace”); and (3) then calculates the Euclidean distance between these points to set the input value for this context information.
- Time: The user chooses “weekday” in the day of week and checks “morning” and “afternoon” in time of day. At the time of inference, the engine: (1) checks the current time from the mobile device clock; (2) formats the data to numerical information used in the fuzzy sets regarding time; and (3) sets the input values for this context information.
- Activity: The user specifies “walking” and “running” and, at the time of inference, the engine: (1) checks the current activities being performed by the user; (2) gets and normalizes the probability values from the specific activities “walking” and “running”; and (3) sets the input value for activity information from the sum of the correspondent probability values.

The above fuzzy rule reaches its maximum activation when applied to the following input values for context data:

- Location: input value from 0 to 800 m from the point registered in the map by the user and the location where the user is at the inferencing time;
- Day of the week: from 1.3 to 5.7, where 24 h are represented in the range from 0 to 1;
- Time of day: from 6 to 12, or from 14 to 17, where 60 min are represented in a scale from 0 to 1;
- Activity: one.

## 4. Computational Implementation and Features

*SituMan* is implemented on the Android platform because of its rich framework and wide acceptance. *SituMan* is composed of: (1) a shared Android service with an API to provide situation awareness to other applications (including *MoodBuster*) that relies on an infrastructure for context data gathering and a situation inference engine from gathered data; and (2) a mobile application (*SituMan* application).

**Figure 4:**
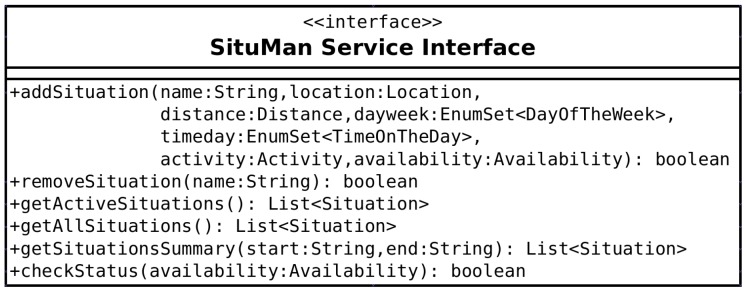
*SituMan* service interface.

*SituMan* contains an Android service implementing an API to enable situation awareness in other applications. To this end, it defines some basic methods, which can be seen in Figure 4, used in both *SituMan* and *MoodBuster* applications for: (i) maintaining situations in the situations database: *addSituation* and *removeSituation* methods; (ii) checking the activated and defined situations, i.e., those situation fuzzy rules with the degree of activation greater than zero: *getActiveSituations* method; (iii) getting all defined situations: *getAllSituations* method; (iv) obtaining the situation summary for a specified time period: *getSituationsSummary* method; and (v) checking the status (i.e., the availability) for a given type of notification (i.e., the user availability to receive a self-assessment request from *MoodBuster*) according to the current user situation: *checkStatus* method. The current user situation is the one having the correspondent fuzzy rule with the highest degree of activation among all defined rules. In this way, by using the *checkStatus* method, *MoodBuster* requests self-assessments at adequate situations, i.e., situations that favour the assessment of the patient. If this is the case, *MoodBuster* will request the patient to express his/her mental status and experiences using the graphical interfaces illustrated in Figure 1. Methods of this API are accessed via Android Interface Definition Language (AIDL) (http://developer.android.com/guide/components/aidl.html), where clients from different applications can access the service and non-primitive data are encapsulated in JSON format. AIDL provides a language to define a programming interface that both client and service agree to using, and they communicate with each other using interprocess communication (IPC), allowing different applications to access a service concurrently.

**Figure 5:**
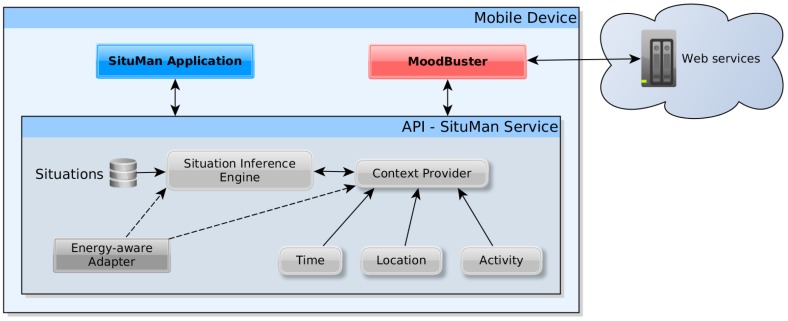
*SituMan* computational architecture.

The *SituMan* architecture integrated with the *MoodBuster* application is illustrated in Figure 5. Web services are used to receive information about mental status and experiences of the patients from the *MoodBuster* mobile application. This information is sent to the Web services to be consulted by professionals via the Web application. The Web application is also used as a direct patient–professional communication channel, providing a means for professionals to perform remote interventions through the *MoodBuster* mobile application, such as the continuous adjustment of medicine prescriptions (e.g., a psychiatrist observing a patient going from a depressed state to a manic state can adjust the medicine accordingly) and behavioural therapeutic feedback to the patient with instructions to patients on how to handle difficult situations (e.g., a psychologist can send descriptions of actions to take when the patient is feeling depressed).

The *Situation Inference Engine* component contains the fuzzy inference engine, which periodically performs the inference process and saves the result in the *Situations Database*. This database is responsible for storing situations defined by users as well as all inferred situations. The *Situation Inference Engine* component is implemented with the *jFuzzyLogic* library [43,44]. *jFuzzyLogic* is an open source fuzzy logic library written in Java programming language used to simplify fuzzy systems developments. In this library, fuzzy rules are written using the Fuzzy Control Language (FCL), which is defined in the IEC 61131 Part 7 specification (http://jfuzzylogic.sourceforge.net/html/manual.html#fcl).

The *Situation Inference Engine* component obtains context data from the *Context Provider* to perform the situation inference process. The *Context Provider* component is responsible for obtaining, formatting and providing context data. The time is obtained from the local clock of the mobile device. As previously mentioned, the user activity is detected using the Google Play Services via the activity recognition API. Context data about the location are obtained from GPS or mobile networks via Google Location Services API, which is also part of Google Play Services (https://developers.google.com/android/reference/com/google/android/gms/location/package-summary). The location information is particularly tricky, because it may become temporally unavailable for several reasons (e.g., GPS satellites are not visible or poor mobile network coverage). For these reasons, *SituMan* implements a temporal decaying function to decrease the confidence in the location data with time. Therefore, the fuzzy inference process performed by the *Situation Inference Engine* component using stale location information will lead to a lower degree of activation of the fuzzy rule.

The *Energy-aware Adapter* component is used to adjust *SituMan* energy consumption depending on the mobile device battery usage level. This battery-preserving approach aims to provide a balance between the frequency of (i) gathering context data and executing the situation inference procedure and (ii) the device energy availability. Shorter time intervals for executing the situation inference will lead to a greater energy consumption. Because of that, situation inference is performed every 3, 6, or 9 min, depending on the device battery level. The context data (time, location, and activity) are collected immediately before executing the situation inference procedure. Nevertheless, the Google Location and Activity services must be kept activated for some time before data retrieval. The longer the service is kept activated, the higher is the probability that the retrieved data will be accurate. Because of that, we also adapt the time between the Location and Activity services’ activation and data retrieval, that can be 35, 45, and 60 s according to the device battery level (longer times will lead to a greater battery consumption). We choose those delay times since 35 s is the minimum value considered secure to receive newer activity data [45].

*SituMan* handles the occurrence of errors, such as the unavailability of location and user activity data. The provision of location information can even be disabled by the user. If the location or user activity data are not available, the *Situation Inference* component will stop the user situation inference. If this holds for 27 min (i.e., three times the largest time interval used to infer the user situation), *SituMan* will inform *MoodBuster* through a callback method that it stopped from inferring the user situation. *MoodBuster* will then switch to the behaviour where the user is required to give his/her mental status and experiences ratings at predefined times. When all context data becomes again available, *SituMan* will restart the user situation inference process, notifying *MoodBuster* that the user situation is available. *MoodBuster* will then switch to the behaviour where the user is required to fill the mental status and experiences ratings depending on his/her current situation. This operation adaptation of the *MoodBuster* is performed using a design pattern called *Component Configurator* [46], which provides a mechanism to (re)configure components in an application dynamically without having to shut down and restart it.

### Proposed Tools for Mental Disorder Treatments

**Figure 6:**
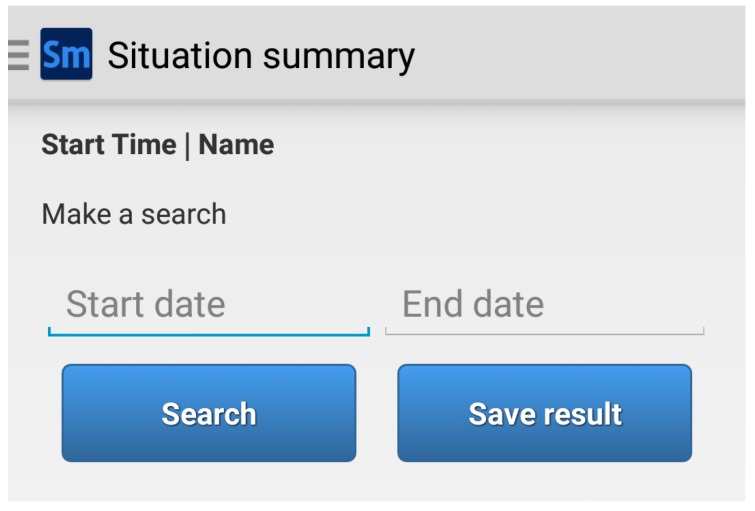
*SituMan* Mobile GUI of the Situations Summary.

*SituMan* and *MoodBuster* applications were designed based on the following requirements: be intuitive and simple to use. In this way, they provide easy to use interfaces, requiring little technical expertise from the users. The *SituMan Application* provides interfaces to: (1) configure situations; (2) check the current user situation; (3) visualize and save a log file of the inferred situations from the *Situations Database*; and (4) guide the user on how to use the application and provide information about *SituMan* and E-Compared research objectives. In addition, the *SituMan Application* provides a summary to search situations experienced by patients in a time period, such as illustrated in Figure 6. All inferred situations are stored in the *Situations Database* and can be consulted by mental health professionals. The summary of situations experienced by patients can show their daily routine and their behavioural patterns to the mental health professional, which provides insight for face-to-face sessions.

**Figure 7:**
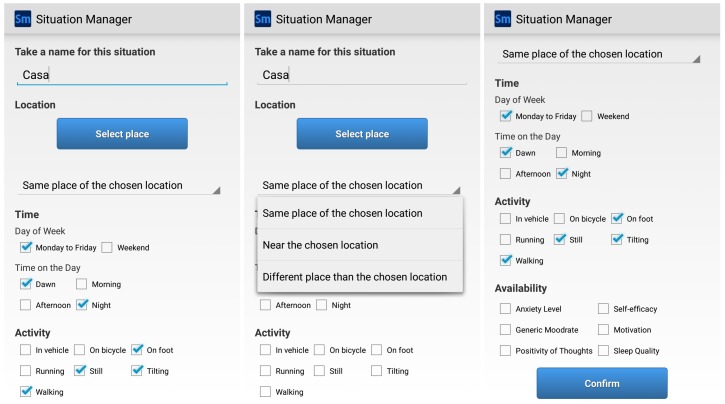
*SituMan* mobile GUI for defining situations.

The *MoodBuster* application was adapted to use the *SituMan Service* to address the issues of recall bias and situation identification by providing situation awareness. The *MoodBuster* application uses the *SituMan Service* API to request patients’ self-assessments based on their current situation (i.e., situation-aware requests for self-assessments) whilst aiming not to be excessively intrusive. These notifications typically request patients to rate their levels of mood, anxiety, sleep quality, positiveness of thoughts, self-efficacy (in achieving tasks) and motivation, as can be seen in Figure 7 (availability option).

An essential requirement for the adoption of *SituMan* by mental health professionals and patients is the provision of an easy to use interface for the definition of situations. For this, the fuzzy logic provides linguistic variables that facilitate this task. Figure 7 shows the *SituMan* interface used to define situations and how it is used to build the situation fuzzy rules that use those variables. Initially, users (typically the patient in collaboration with their mental health professional) define the name and context information that characterize the situation, by providing a location, day of the week, time of day and activity.

Once the mental health professional and patient have defined situations of interest, they have to set the types of notifications (i.e., request for self-assessment) that the patient is available to receive for each defined situation. The assessments used were: anxiety level, mood rate, positivity of thoughts, self-efficacy, motivation and sleep quality. Since the situations of interest and assessments have been defined, *SituMan* works properly and users need to return to this task only in the event of managing situations (e.g., add, remove, or redefine situations).

With this mapping of assessments to situations, *SituMan* can respond to requests of the *MoodBuster* application to display a certain assessment. To this end, the *MoodBuster* application will query the *SituMan* service using the *checkStatus* method in the service API. This method will respond with a message that indicates whether or not the assessment may be presented to the patient, given the currently inferred situation.

## 5. Experimental Evaluations

In this section, we describe two experience-sampling studies in which participants used *SituMan*. The first experiment aimed to evaluate the *SituMan* usability and the user satisfaction regarding the definition and identification of situations. The second experiment aimed to estimate the accuracy of the fuzzy inference engine to identify situations.

### 5.1. Experiment 1: User Satisfaction

For any technical add-on to mental health treatment, user acceptability and experience are of great importance. This especially holds true where users may perceive technology to have a potential impact on their privacy.

#### 5.1.1. Methodology and Participants

We recruited participants from the Institute for Systems Engineering and Computers, Technology and Science (INESC TEC) and the University Institute of Maia (ISMAI) through project meetings and presentations. Volunteers were healthy non-depressed individuals who knew the project objectives and had knowledge of the research areas of intersection: mental health treatments, as well as information and communication technologies (ICT). We chose participants with these characteristics as they are the most likely to identify problems for the use of the proposed system in mental health. All participants were required to have an Android smartphone and to have good knowledge of the English language, because the mobile application and the questionnaire were produced in this language. All subjects gave verbal consent and were fully committed to participate in the study.

We had a total of nine participants (seven male) that answered the questionnaire: five psychologists (they hold a Ph.D. or were Ph.D. candidates in psychology) and four ICT researchers (similarly, they hold a Ph.D. or were Ph.D. candidates in the informatics area). All participants were Portuguese citizens and fluent in the English language. They were aged as follows: two were less than 26 years old, one 26 to 30, two 36 to 40, three 41 to 45, one 51 to 55. Prior to the study, the participants were instructed on use of *SituMan*, by means of: online documentation on a web-site, oral explanations and a live demonstration of realistic usage scenarios. Any follow-on questions subjects had regarding the operation of the *SituMan* were responded to by email or direct personal contact.

All subjects answered an online questionnaire after at least seven days of using the application. This time period was deemed necessary for users to learn the proposed functions and behaviour of *SituMan* and to give their opinion about their usage experience. We believe this time period was also sufficient for users to experience different daily routine situations and to evaluate whether *SituMan* was able to correctly infer their situations. Questionnaires were anonymous, but participants were requested to provide nationality, age and gender information. The questionnaire had nine questions to be answered on a five-level Likert scale [47] with responses: very bad, bad, regular, good and very good.

#### 5.1.2. Results

**Figure 8:**
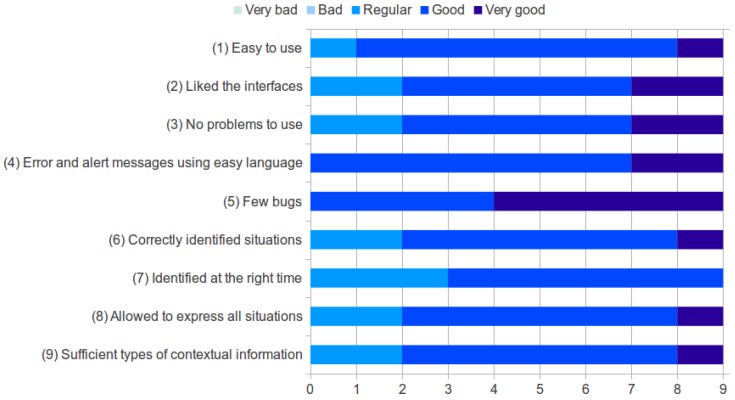
Results from the questionnaire.

Figure 8 summarizes all results from the nine questions. The first five questions of the questionnaire evaluated the general usability of *SituMan*. All participants considered *SituMan* easy to use (Question 1), liked to use the interfaces provided to perform the functionalities (Question 2) and had no problem learning how to use *SituMan* (Question 3). All subjects answered “very good” or “good” when asked if error and alert messages are expressed in a simple and easy to understand language (Question 4). Moreover, all users also considered that few errors (bugs) caused by the *SituMan* occurred (Question 5).

To evaluate the situation inference engine, the participants were asked if *SituMan* correctly identified their situations (Question 6) and whether *SituMan* identified their situations at the right time (Question 7). One respondent answered the “very good” option, and all remaining marked the “regular” or “good” options for Question 6. All respondents answered Question 7 with “regular” or “good”. The participants were also asked if *SituMan* allowed them to express all of their situations (Question 8) and if the types of contextual information (location, day of the week, time of day and activity) used to express the situations were sufficient (Question 9): in both questions, seven users responded positively (“good” and “very good”) and two of them answered the regular option; all responses were the same in both questions.

### 5.2. Experiment 2: Accuracy of Situation Identification

The second experiment aimed to check the accuracy of *SituMan* in identifying situations by requesting users to confirm whether the identified situation was indeed correct.

#### 5.2.1. Methodology and Participants

The participants of the second experiment were recruited from the Laboratory of Intelligent Distributed Systems at the Federal University of Maranhão and INESC TEC. Similar to the first experiment, volunteers used the *SituMan* during exactly seven days. All participants were required to have an Android smartphone. A total of 12 subjects (six female) participated in this user experiment. Regarding the nationality, eight subjects were Brazilian and four Portuguese citizens. Participants were aged between 23 and 51 years (average = ≈32.91, SD = ≈9.09).

Prior to the study the participants were instructed on how to use *SituMan* by means of: oral explanations and sample scenarios demonstrated by the authors. Moreover, the subjects had their questions about *SituMan* operation answered via email. Participants were requested to define at least two of their daily routine situations in the *SituMan*.

**Figure 9:**
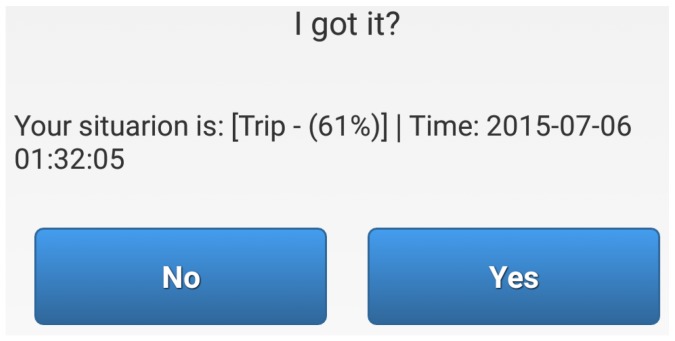
*SituMan* mobile GUI for requesting confirmations from participants.

The version of *SituMan* used in this second experiment had a feature to prompt the user with a notification whenever a new situation is identified. These notifications were used to ask participants whether the identified situation was correct by prompting a “yes” or “no” answer. An example of the notification is shown in Figure 9: “trip” is the name of the situation; “61%” represents the chance that the situation is happening (i.e., degree of activation of the situation fuzzy rule), in which the symbol % was used to facilitate the understanding of the user; users were also informed of the time at which the situation was identified. The application logged all user answers, situation definitions and their time-stamps. We requested the users to send us their logs after using the application.

#### 5.2.2. Results

Table 2 presents the results extracted from the logs. Defined situations shows the amount of situations configured by the user; Correct and incorrect represent the number of correct and incorrect situations confirmed by the participants, i.e., those confirmed with a “yes” and “no” answer. The percentage of correct situations is presented in relation to all of the confirmed situations.

The notifications generated for requesting confirmations from participants were overwritten by newer notifications, i.e., only the most current situation inferred could be confirmed as correct or incorrect by the participants. This explains the difference among situations confirmed by users as participants were not always available to respond to these notifications.

### 5.3. Discussion

There is extensive literature on how mobile technologies can support the treatment of mental disorders [3], and the validity of computer-assisted self-assessments in depression treatments is a topic of significant interest [48]. The advantages and disadvantages in using mobile technologies for psychosocial interventions are well known [49], and one of these is the absence of contextual information in EMA/Is. The evaluation presented in this paper focuses on demonstrating the feasibility of applying our proposed model for situation definition and real-time inference that leads to a more adequate and unobtrusive patient assessment, as well as more suitable interventions.

The first experiment showed that *SituMan* usability was well evaluated. This result is important to show that: the usability was not a factor that negatively influenced the use of the solution, and the features offered by the application were easily used. The evaluation also showed the efficiency and reliability of the inference engine to identify situations, because the user feedback was that *SituMan* identified the user situations well and at the right time. Since the inference engine can identify situations correctly and at the right time, the notifications (requests for self-assessment) are made by the *MoodBuster* at adequate moments. We believe that none of the participants provided the answer “very good” in Question 7, an expected outcome, because of the refresh rate of the context data. By using a battery-preserving approach, there is a variable delay for updating context data and performing the situation inference process, i.e., to identify the most current situation (see Section 4). The maximum delay time is about nine minutes, and we feel this is an acceptable delay in most scenarios where the context information is used to prompt a user and to save identified situations in the summary.

The outcome of Questions 8 and 9 confirms a good acceptance of the *SituMan* approach to allowing participants to build situations. Nevertheless, we plan on making the definition of situations easier by introducing the concept of situation templates, which enable the reuse of situation settings. A situation template is a default situation setting with context data partially customized, which reduces the effort for the user to configure situations. For example, a user only needs to specify the location information in order to create a situation, having previously been defined by default values for other types of context data. In addition, we are also investigating the benefits and drawbacks of allowing the use of co-location information (i.e., people nearby the patient) to compose a situation definition. This type of contextual information is important to check whether patients take part in social interaction, which is a construct of great significance in depression treatments.

The second experiment also showed that the situation inference engine proved to have a good accuracy to identify situations. As can be seen in Table 2; the participants configured from two to six situations during the seven days. Most of the inferred situations were confirmed as correct by the participants: 490 confirmations, 451 correct and 39 incorrect. Therefore, this accuracy evaluation revealed a good situation recognition capability as evidenced by the large proportion of true positive situations (i.e., those confirmed as correct by participants) to known false positive situations (i.e., those confirmed as incorrect by participants): ≈92.04% of success.

An important concern is related to the user privacy, since the tracking of mental status and experiences are made by the proposed system. It is worth highlighting that there is an agreement between the mental health professional and the patient, in which the patient agrees with the discovery of that information during his/her treatment to enrich the monitoring made by the mental health professional. In addition, the *SituMan Service* API provides methods to retrieve the history of inferred situations. However, the current *MoodBuster* application does not send it to the Web services. On the other hand, in a future release of *MoodBuster*, we plan to add a function that enables the periodical transmission of the patient inferred situations to the Web services. However, this may lead to a privacy concern, and we also plan to allow the patient to control this functionality by switching it on/off as he/she desires.

## 6. Conclusions

In this paper, we describe *SituMan*, a mobile system that makes use of the sensors commonly included in most mobile platforms and a fuzzy inference engine to infer user situations. The system was used to enhance *MoodBuster*, an existing app that collects ecological momentary assessments with contextual information such that the timing of response requests can be tailored to the user’s current situation. This approach is a first step in truly addressing the issues of recall bias and situation identification in the scope of depression treatments by allowing self-reports to be requested immediately after a troublesome (or otherwise interesting) situation is identified as having occurred, thus reducing obtrusiveness. *SituMan* can also provide new insights for mental health professionals by summarizing the situations experienced by the patient, further allowing the correlation of situation information with patient self-report data within the same time frame. The correlation can be used by the mental health professional to identify problematic or good situations experienced by the patients.

The first experiment with *SituMan* demonstrated good user satisfaction. Importantly, users with a psychology background showed good receptivity towards the proposed solution. Therefore, the initial results showed that the solution has feasibility to be evaluated for real depressive patients. In future work, we will perform a more extensive and thorough evaluation and obtain results from real use scenarios and in a real patient population.

Regarding the accuracy evaluation in the second experiment, we acknowledge that the chosen test protocol did not allow for the analysis of false negatives, i.e., those defined situations that the participants experienced, but the *SituMan* failed to identify. We plan on investigating the false negative rate of the inference engine by performing scripted experiments with users in which users follow a protocol that prescribes the situations that must be “visited” by the user whilst the latter is being monitored or performs self-reports on when each of the situations described in the protocol occurs. These findings can then be compared with the situations identified by the inference engine to obtain information on false negatives.

## Figures and Tables

**Table 1:** Membership Functions for Weekend and Night Fuzzy Sets.

Fuzzy Set	Membership Function
**Night** (Night1 ∪ Night2)	$\mu \left(x\right)=\left\{\begin{array}{cc} 0, & \mathrm{if}\;3<x\leq 19;\\ \frac{x-19}{2}, & \mathrm{if}\;19<x\leq 21;\\ \frac{3-x}{2}, & \mathrm{if}\;1<x\leq 3;\\ 1, & \mathrm{if}\;21<x\leq 1. \end{array}\right.$
**Weekend** (Weekend1 ∪ Weekend2)	$\mu \left(x\right)=\left\{\begin{array}{cc} 0, & \mathrm{if}\;1.3<x\leq 5.7;\\ \frac{x-5.7}{0.6}, & \mathrm{if}\;5.7<x\leq 6.3;\\ \frac{1.3-x}{0.6}, & \mathrm{if}\;0.7<x\leq 1.3;\\ 1, & \mathrm{if}\;0.7\geq x>6.3. \end{array}\right.$

**Table 2:** Results from the accuracy evaluation.

Participant	Defined	Correct	Incorrect
**1**	3	45 (100%)	0
**2**	3	28 (≈90.32%)	3
**3**	4	42 (100%)	0
**4**	5	33 (≈86.84%)	5
**5**	3	38 (≈90.47%)	4
**6**	6	54 (100%)	0
**7**	5	60 (≈86.95)	9
**8**	5	73 (≈86.90)	11
**9**	4	14 (≈77.77%)	4
**10**	4	10 (≈90.90%)	1
**11**	3	43 (≈97.72%)	1
**12**	6	11 (≈91.66%)	1

## Abbreviations

The following abbreviations are used in this manuscript:

AIDL	Android Interface Definition Language
API	Application Programming Interface
EMA	Ecological Momentary Assessment
EMI	Ecological Momentary Intervention
EMA/I	Ecological Momentary Assessment and Intervention
FCL	Fuzzy Control Language
GUI	Graphical User Interface
INESC TEC	Institute for Systems Engineering and Computers, Technology and Science
ISMAI	University Institute of Maia
*SituMan*	Situation Manager
